# Error detection using a multi‐channel hybrid network with a low‐resolution detector in patient‐specific quality assurance

**DOI:** 10.1002/acm2.14327

**Published:** 2024-03-15

**Authors:** Bing Yan, Jun Shi, Xudong Xue, Hu Peng, Aidong Wu, Xiao Wang, Chi Ma

**Affiliations:** ^1^ School of Instrument Science and Optoelectronics Engineering Hefei University of Technology Hefei China; ^2^ Department of Radiation Oncology The First Affiliated Hospital of University of Science and Technology of China Hefei China; ^3^ School of Computer Science and Technology University of Science and Technology of China Hefei China; ^4^ Department of Radiation Oncology Hubei Cancer Hospital, TongJi Medical College Huazhong University of Science and Technology Wuhan China; ^5^ Department of Radiation Oncology Rutgers‐Cancer Institute of New Jersey Rutgers‐Robert Wood Johnson Medical School New Brunswick New Jersey USA

**Keywords:** deep learning, error detection, intensity modulated radiation therapy, multi‐channel hybrid network, patient‐specific QA

## Abstract

**Purpose:**

This study aimed to develop a hybrid multi‐channel network to detect multileaf collimator (MLC) positional errors using dose difference (DD) maps and gamma maps generated from low‐resolution detectors in patient‐specific quality assurance (QA) for Intensity Modulated Radiation Therapy (IMRT).

**Methods:**

A total of 68 plans with 358 beams of IMRT were included in this study. The MLC leaf positions of all control points in the original IMRT plans were modified to simulate four types of errors: shift error, opening error, closing error, and random error. These modified plans were imported into the treatment planning system (TPS) to calculate the predicted dose, while the PTW seven29 phantom was utilized to obtain the measured dose distributions. Based on the measured and predicted dose, DD maps and gamma maps, both with and without errors, were generated, resulting in a dataset with 3222 samples. The network's performance was evaluated using various metrics, including accuracy, sensitivity, specificity, precision, F1‐score, ROC curves, and normalized confusion matrix. Besides, other baseline methods, such as single‐channel hybrid network, ResNet‐18, and Swin‐Transformer, were also evaluated as a comparison.

**Results:**

The experimental results showed that the multi‐channel hybrid network outperformed other methods, demonstrating higher average precision, accuracy, sensitivity, specificity, and F1‐scores, with values of 0.87, 0.89, 0.85, 0.97, and 0.85, respectively. The multi‐channel hybrid network also achieved higher AUC values in the random errors (0.964) and the error‐free (0.946) categories. Although the average accuracy of the multi‐channel hybrid network was only marginally better than that of ResNet‐18 and Swin Transformer, it significantly outperformed them regarding precision in the error‐free category.

**Conclusion:**

The proposed multi‐channel hybrid network exhibits a high level of accuracy in identifying MLC errors using low‐resolution detectors. The method offers an effective and reliable solution for promoting quality and safety of IMRT QA.

## 1 INTRODUCTION

Intensity Modulated Radiation Therapy (IMRT) is a critical modality in radiation therapy, offering significant dose modulation capabilities to deliver high doses to the tumor while minimizing dose to surrounding organs.^1^ Since numerous control points are used in IMRT for dose modulation, ensuring patient safety and treatment efficiency necessitates thorough quality assurance (QA) procedures.^2^ QA in IMRT encompasses several steps, including transferring the clinal treatment plan to a measurement device, delivering the planned dose to the measurement device, and analyzing the measured dose compared to the planned dose. Gamma analysis is widely utilized in clinical practice to assess the accuracy of treatment plans,^3^ which takes both dose deviations and distance‐to‐agreement between measurements and calculated points into account. Typically, gamma analysis employs criteria such as a 3% dose deviation and 2 mm spatial distance, with a 10% dose threshold. A gamma index exceeding 95% indicates the clinical acceptability of the treatment plan.^4^

Although gamma analysis is a commonly used QA method in clinical practice to determine the accuracy of treatment delivery, clinical physicists are unable to determine the cause of the errors if the QA results are deemed faulty.^5^ To address this problem, a combination of radiomics and machine learning was employed to identify delivery errors in IMRT QA. This approach involved extracting numerous quantitative features (e.g., shape, texture, intensity) from dose difference (DD) maps or gamma maps generated by electronic portal imaging device (EPID). Subsequently, machine learning algorithms, such as logistic regression or support vector machines, were applied to these radiomic features for error classification in IMRT QA. Wootton et al. utilized radiomics analysis to classify multileaf collimator (MLC) random and systematic errors based on the gamma distribution.^6^ Nyflot et al. employed machine learning methods to analyze gamma images generated from EPID to identify different MLC errors.^7^ Ma et al. also utilized machine learning methods to analyze structural similarity index measure (SSIM) index generated from EPID images to identify delivery errors, including MLC and monitor unit (MU) errors.^8^ Leveraging the remarkable success of deep learning in machine vision classification tasks, another approach involved utilizing Convolutional Neural Networks (CNN) for feature extraction from DD maps generated from 3D detectors to facilitate error detection. Kimura et al. employed DD maps generated from Delta4 phantom (ScandiDos, Sweden) and applied CNN to predict two types of MLC errors.^9^ Both methods yielded superior results in error detection compared to traditional gamma analysis.

In previous research,^6^, ^7^, ^8^ the DD maps or gamma maps used for error detection were obtained from either EPID or 3D detectors. Although these two detectors have been widely applied in clinical practice, 2D dosimeters such as 2D diode arrays or 2D ion chamber arrays are more commonly used for patient QA.^10^ Therefore, the direct utilization of 2D arrays for identifying machine delivery errors holds clinical significance. However, due to the superior spatial resolution of EPID compared to 2D detector arrays, and the high sensitivity of EPID in patient‐specific QA, the application of low‐resolution 2D arrays presents a considerable challenge for detecting errors in QA.^11^, ^12^ Therefore, one aim of our research is to apply deep learning methods in conjunction with a low‐resolution 2D array for MLC error detection.

Furthermore, it is important to note that previous research efforts utilizing CNN methods for MLC error identification in patient‐specific QA only used a single image type as input, either the DD map or the gamma image. Kimura et al. showed that using the DD map as input yielded superior results in CNN predictions compared to the gamma images, though this research was based on 3D detectors and did not involve a 2D array.^9^ Wolfs et al. investigated the effect of many different dose comparison methods on the performance of AI models for error detection in pre‐treatment QA.^13^ In specific‐patient QA, the DD map is highly sensitive in high‐gradient dose regions, where small spatial discrepancies can result in significant DDs. However, these discrepancies may not be caused by MLC errors but rather human operation errors or set‐up errors, such as detector positioning errors. Although gamma images are not highly correlated with the spatial positions of failed points, they align better with clinical application guidelines.^4^ Therefore, the other aim of this study is to determine the types of images to use as input for deep learning when utilizing a low‐resolution 2D array, particularly to combine DD maps and gamma images as multi‐channel inputs to enhance the performance of the deep learning network.

In this study, we developed a novel deep‐learning network for MLC error detection using a low‐resolution 2D array. This network utilized DD maps and gamma maps as multi‐channel inputs to improve network performance. The training parameters and source code can be found on Github (https://github.com/shijun18/MLC_CLS).

## 2 METHOD

### 2.1 Treatment plan and delivered dose measurement

In this study, 68 IMRT plans of patients treated in our hospital were selected. The anatomic sites of the plans include head and neck, chest, abdomen, and pelvis. There were 358 beams in all plans, and the number of beams per plan ranged from 3 to 13. All treatment plans used 6MV photon with a dose rate of 600MU/min, designed using the Monaco (Version 5.11.02, Elekta, Sweden) treatment planning system (TPS) and the Monte Carlo dose algorithm with the sliding window technique of fixed‐gantry IMRT and performed on the Elekta Synergy linear accelerator with a 1 cm width MLC at the isocenter.^14^

The perpendicular field‐by‐field method was utilized to measure these patient‐specific IMRT plans. All IMRT plans were transferred to be delivered to the 2D ion chamber array (PTW seven29, PTW, Germany), with the gantry angle at 0 degree, and the dose was recalculated using TPS. In this way, the calculated dose is in the same plane as the detector. IMRT QA plans were delivered to the 2D ion chamber array with gantry at 0 degrees for all beams. The measuring device was calibrated using a 10×10 cm field before measurement.

### 2.2 MLC error simulation

To facilitate a comparative analysis with previous literature and substantiate the effectiveness of our method,^6^, ^7^, ^8^ four types of MLC position error plans including shift, opening, closing, and random were simulated in this study. The types of errors are described below.

Shift error: For each control point of the beams, all MLC leaves were shifted by 2 and 3 mm in the same direction, resulting in identical field sizes and a 2 and 3 mm shift relative to error‐free plans.

Opening error: For each control point of the beams, all MLC leaves were modified by 1 and 2 mm in the opposite direction away from the beam axis. Compared with the error‐free plan, this error plan corresponds to an expansion of the field size by 2 and 4 mm in the *X*‐direction for all control points of the beams.

Closing error: To simulate a closing error, the positions of all MLC leaves were adjusted by 1 and 2 mm towards the beam axis for each control point of the beams, resulting in 2 and 4 mm shrinking in the field size of all control points compared to the original error‐free plan.

Random errors: For each control point of the beams, all MLCs move randomly according to a Gaussian distribution, with standard deviation δ = 1 and 2 mm of errors.

To acquire the predicted dose of these MLC leaf error plans to the same plane as the 2D ion chamber array, error‐free IMRT plans were exported from TPS as DICOM RT‐plan files. The position of all control points of the MLC leaves in the RT‐plan files were modified to the specified position using an in‐house program in MATLAB (Version R2016a, Math Works, USA), and then the modified RT‐plan files were re‐imported into TPS and recomputed to generate predicted dose.

**FIGURE 1:**
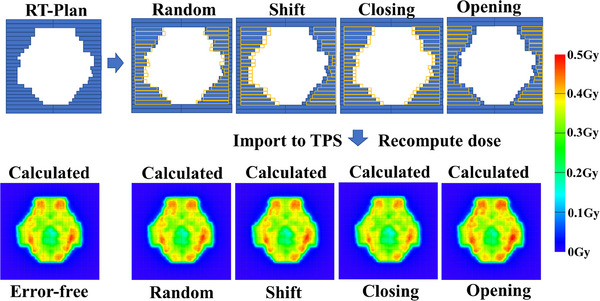
Examples of MLC positions and predicted doses with four types of introduced errors. The blue solid line represents the MLC positions in the error‐free plan. The yellow dashed line represents the MLC positions in the error plan. MLC, multileaf collimator.

Measured dose maps for the error‐free plan were acquired via delivered dose measurement, while prediction maps for both the error‐free plans and eight MLC leaves error types of plans were obtained through error simulation, as shown in Figure 1.

### 2.3 Data processing

**FIGURE 2:**
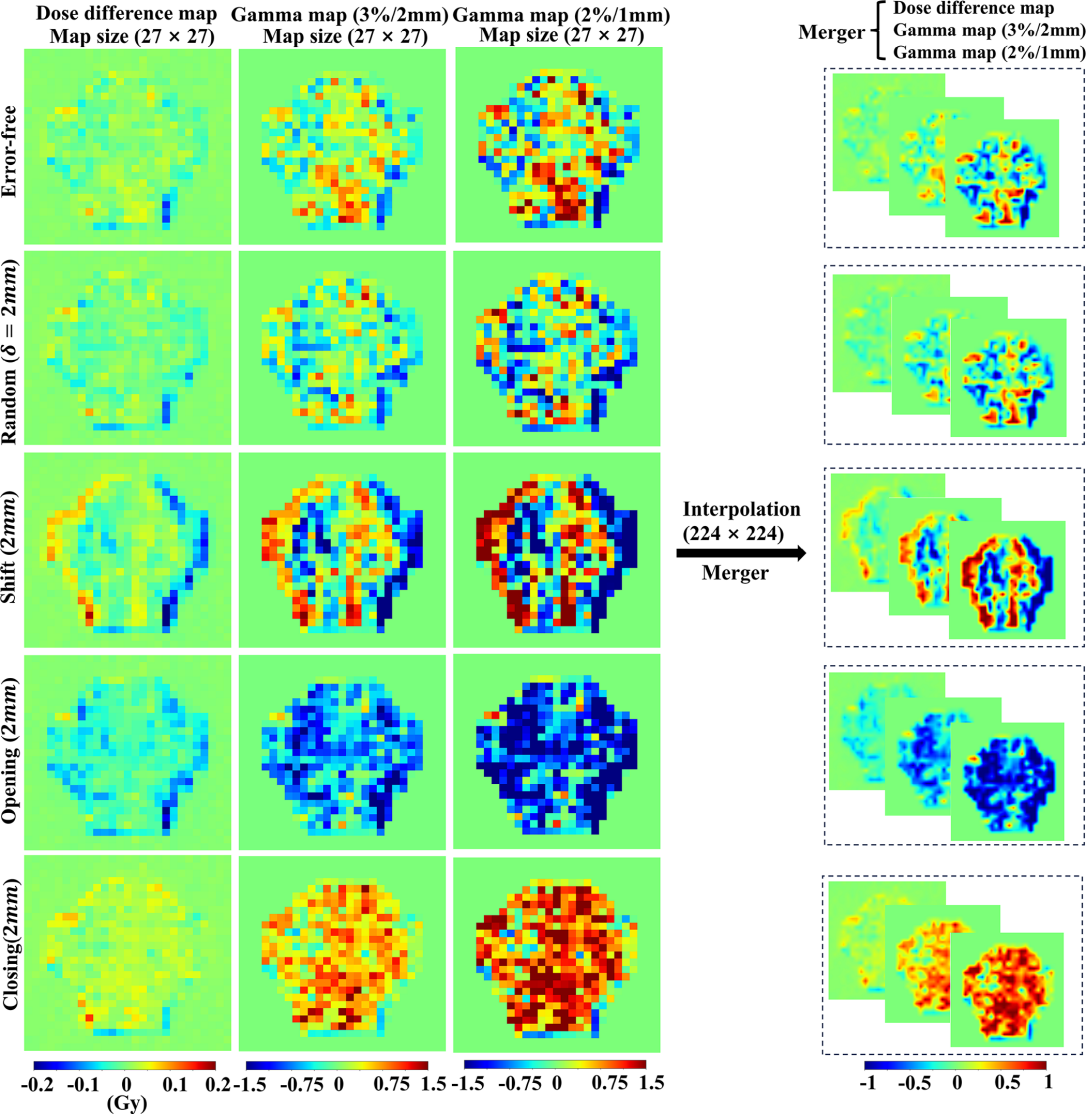
Examples of dose difference maps and gamma maps under two different criteria for a single beam. From top to bottom, the difference maps correspond to the following scenarios: error‐free, random error, shift error, opening error, and closing error. These maps were generated based on predicted dose distributions with and without errors, where the reference dose distribution is the predicted dose distribution with and without errors, and the measured distribution corresponds to the error‐free measured dose distribution.

Three hundred fifty‐eight measured dose maps of error‐free plans and 3222 prediction dose maps were obtained through QA measurement and simulation of MLC leaves error plans. The predicted dose maps include 358 error‐free plans and 2864 error plans, with 716 plans for each of the four error types: random, opening, closing and shift errors. These maps were processed to obtain DD maps and gamma maps, which were used as input to the deep learning algorithm for classifying MLC leaves delivery errors. Kimura's method^15^ was employed to ensure consistency in processing the DD maps. The processing steps for the gamma maps were similar, except that the gamma maps were evaluated using two passing rate criteria of 3%/2 mm and 2%/1 mm, with a dose threshold of 10% and global normalization.^4^ Positive and negative symbols were introduced in the gamma evaluation to distinguish hot and cold spots in the predicted and measured dose maps. Specifically, negative gamma values represented cold spots, while positive gamma values represented hot spots. Finally, a normalization process was conducted to use the gamma values as the network input. The gamma values were subjected to thresholding with an upper limit value of 1.5 and a lower limit value of −1.5, then a linear scaling was applied to normalize the values to the range of −1 to 1, following the method of Kimura.^9^ Before training, all pre‐processed images were merged and linearly interpolated to 224 × 224 ×3. We interpolated the size of images from 27 × 27 to 224 × 224 to improve the model's performance for two reasons. The first reason is that the architecture of the network in our study is very deep and contains multiple downsampling processes. For each downsampling process, the image size undergoes a 50% reduction. Employing a 27 × 27 image size would entail a considerable loss of spatial information within the feature map after several downsampling processes, adversely affecting the model's overall performance. The second reason is based on Wolfs's research, which indicates that higher‐resolution images can enhance the classification performance of deep learning networks.^13^ It is recommended to use an image resolution of 128 × 128 with a pixel size of 0.7 mm × 0.7 mm for the input of the classification model. As the two‐dimensional low‐resolution detector (PTW seven29, PTW, Germany) used in our study has a resolution of only 27 × 27 (with a pixel size of 1 × 1 cm for the original dose images), to improve the performance of the model, we resized the images to 224 × 224 with a pixel size of 1.2 mm × 1.2 mm. Gamma maps were processed using Pygamma, a Python package available on GitHub (https://github.com/christopherpoole/pygamma).^16^ Figure 2 illustrates a typical processed DD map and gamma map.

### 2.4 Network architecture

**FIGURE 3:**
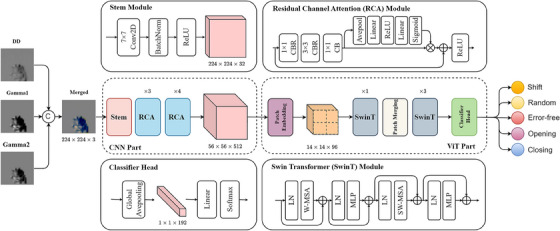
Overall architecture of our proposed HybridNet consists of CNN and ViT parts. CBR represents the successive operator cluster of Convolution‐Batch Normalization‐ReLU, while LN, patch embedding and merging, and MLP are the standard modules in Swin Transformer. W‐MSA and SW‐MSA denote multi‐head self‐attention modules with regular and shifted windows, respectively. CNN, convolutional neural networks; LN, layer normalization; MLP, multi‐layer perceptron.

As shown in Figure 3, the proposed method was a hybrid structure that combined the CNN and Vision Transformer (ViT).^17^ There were two considerations behind this design: On one hand, due to the constraints of the local receptive field, the existing CNN‐based classification methods^18^, ^19^, ^20^, ^21^, ^22^ had limited performance in MLC error identification. On the other hand, although most ViT‐based structures^17^, ^23^, ^24^ could model global spatial dependencies, the data‐hungry property of these methods led to the need for large‐scale labeled data, which was an insurmountable challenge in our task. Therefore, from the perspective of combining the advantages of the two structures, we proposed a novel multi‐channel hybrid network (HybridNet‐MC) for MLC error identification. HybridNet‐MC comprised CNN and ViT structures connected in series, where the former extracted the semantic features of the merged image, and the latter learned global dependencies to obtain high‐quality discriminative features and projected them into the decision space.

Specifically, the CNN part of the network consisted of a stem module and multiple Residual Channel Attention (RCA) modules. The stem module performed feature encoding on the input image through a successive Convolution‐Batch Normalization‐ReLU (CBR) operator cluster with a kernel size of (7,7), resulting in a low‐level feature map of (224, 224, 32) resolution. Subsequently, multiple RCA modules extracted high‐level semantic features with lower spatial resolution and larger feature dimensions from these low‐level feature maps. To reduce the computation cost, the RCA module adopted a bottleneck structure, expanding the feature dimension by the CBR operator cluster with a kernel size of (1,1). In the ViT part, the Swin Transformer (SwinT) module was employed to model the global context dependencies to enhance semantic representation. Unlike the standard ViT structure, the SwinT module introduced a non‐overlapping local window mechanism and achieved cross‐window information interaction through a shift operation, leading to lower computational complexity. During the decision‐making stage, the discriminative features obtained from the network were mapped to predicted probabilities by the classifier head. The predicted result corresponded to the error category with the highest probability. Compared with pure CNN and ViT structures, our method could extract local and global semantic features and eliminate the data‐hungry attribute, making it more suitable for MLC error identification. The experimental results also proved the effectiveness of the proposed method.

### 2.5 Comparison models

We employed two types of networks to compare the performance of HybridNet‐MC and validate the impact of multi‐channel input and hybrid network architecture on the model. Three single‐channel hybrid models (HybridNet‐SC) were implemented to investigate the effect of multi‐channel input on the model. These networks were named HybridNet‐DD, HybridNet‐Gamma32, and HybridNet‐Gamma21 based on the input image types (DD maps, 3%/2 mm standard gamma maps, and 2%/1 mm standard gamma maps, respectively). In the same dataset, pure CNN and ViT models, such as ResNet18^18^ and Swin Transformer,^17^ were trained and tested to study the advantages of the hybrid architecture. To ensure experimental reliability, these networks adopted the same optimization method and training strategy as HybridNet‐MC.

### 2.6 Implementation details

**FIGURE 4:**
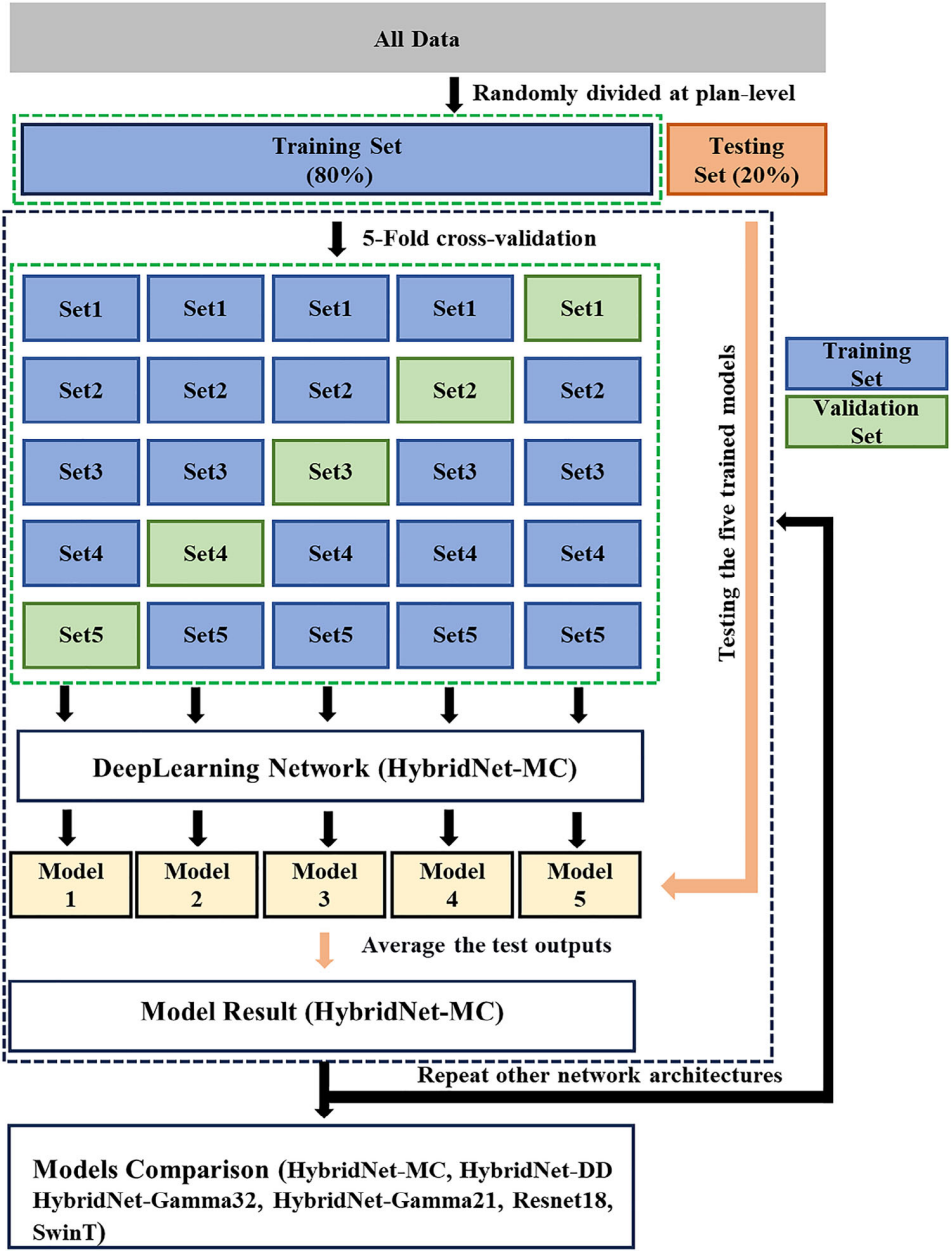
The process of model training and evaluation. A five‐fold cross‐validation approach was implemented during model training, resulting in five trained models. The test dataset was evaluated separately with each of the five trained models, and the mean value of the outputs was the model result. To mitigate the overfitting problem, an early stopping strategy was utilized with a tolerance of 50 epochs to search for the best model within 150 epochs.

The dataset was randomly split into training and testing sets following an 80:20 in the training and evaluation stages. This division was carried out at the plan level to ensure a comprehensive assignment of all DD maps and gamma maps for beams within the same plan to the training and testing sets. A five‐fold cross‐validation approach was implemented during model training, resulting in five trained models. The test dataset was evaluated separately with each of the five trained models, and the mean value of the outputs was the model result, as shown in Figure 4. PyTorch, the popular deep learning framework, was used to implement the baselines and the proposed method. Each fold of all models was trained from scratch using 1 NVIDIA A100 graphics processing unit (GPU) with 40 GB of memory. The cross‐entropy loss was adopted as the optimization function, using the AdamW^25^ optimizer with an initial learning rate of 1e‐5 and a weight decay of 1e‐4. In particular, the cosine annealing strategy^26^ was applied to adjust the learning rate during the training process. The default batch size was 32, and the input sizes for the merged and single‐channel images were 224 × 224 × 3 and 224 × 224 ×1 pixels, respectively. To mitigate the overfitting problem, an early stopping strategy was utilized with a tolerance of 50 epochs to search for the best model within 150 epochs.

Moreover, online data augmentation was conducted, including random erasure, affine, rotation, flipping, and color jitter. Although random erasure and color jitter may alter the physical properties of DD images, numerous studies have demonstrated that these data augmentation methods do not adversely affect the model's performance.^27^, ^28^, ^29^, ^30^ Dropout prevents overfitting by discarding (both hidden and visible) units of the CNN with a probability of p. Inspired by dropout, random erasure is somewhat similar to performing dropout on the image level.^27^ Random erasure involves locally obscuring parts of an image, compelling the model to learn more diverse and descriptive features, thereby preventing the model from overfitting to specific visual characteristics. Similar to our work, Nakamura^28^ created and evaluated deep learning models for the detection and classification of Transmission Factor (TF) and Dosimetric Leaf Gap (DLG) errors in volumetric modulated radiation therapy (VMAT). This study also employed random erasure for data augmentation on dose‐difference images, demonstrating that random erasure does not affect the model's performance. Color jitter is a commonly used data augmentation technique in computer vision and image processing, aimed at increasing the diversity of training data and enhancing the robustness of deep learning models through subtle changes to the colors of images.^29^ Feng et al. employed a siamese network, combining CT images and 3D dose distributions, to predict radiation pneumonitis. In their study, data augmentation included random flipping, rotation, contrast adjustment, color jitter, and affine transform, achieving a prediction accuracy of 0.818, demonstrating the feasibility of applying color jitter data augmentation to dose image datasets.^30^ Based on this research, we used the color jitter technique in our data augmentation. It should be noted that, in our study, color jitter only transformed the contrast and brightness of the images. This alteration represented a global change, and the differences between each pixel were preserved.

The training parameters and source code can be found on Github (https://github.com/shijun18/MLC_CLS).

### 2.7 Model evaluation

The One versus All methodology was utilized to compute the Receiver Operating Characteristic (ROC) curves and the Area Under the ROC Curve (AUC) to assess the networks' classification effectiveness.^31^ The AUC ranges between 0 and 1, with higher values indicating superior classification capabilities. To further evaluate the classification performance of the networks, several metrics were computed, including accuracy, precision, sensitivity, F1‐score, and a normalized matrix which can help visually examine the relationship between the predicted results of the different networks and the true labels. Furthermore, the t‐SNE method was applied to reduce the dimensionality of the network's feature maps to a two‐dimensional space, facilitating the visualization of classification errors across diverse networks.^32^

## 3 RESULTS

### 3.1 Classification results of HybridNet‐MC

Table 1 presents the precision, accuracy, sensitivity, specificity, and F1‐scores of the hybrid network for the five catalog classifications in the test dataset of five‐fold cross‐validation. The mean values for the precision, accuracy, sensitivity, specificity, and F1‐score were 0.87, 0.85, 0.85, 0.97, and 0.89, respectively. The results showed that the metrics from each iteration of the cross‐validation consistently exhibited similarity, indicating that HybridNet possessed exceptional stability and robustness in the variations in the input data.

**TABLE 1:** The precision, accuracy, sensitivity, specificity, and F1‐score of the HybridNet‐MC model, which employed the dose difference maps and two criteria gamma maps (3%/2 mm, 2%/1 mm), were evaluated through five‐fold cross‐validation.

		Value					
Parameter	Error type	Fold 1	Fold 2	Fold 3	Fold 4	Fold 5	All folds
Accuracy	Shift Error	0.91	0.89	0.89	0.88	0.89	0.89
”	Random Error	0.91	0.89	0.89	0.88	0.89	0.89
”	Error‐free	0.91	0.89	0.89	0.88	0.89	0.89
”	Opening Error	0.91	0.89	0.89	0.88	0.89	0.89
”	Closing Error	0.91	0.89	0.89	0.88	0.89	0.89
Precision	Shift Error	1.00	1.00	1.00	1.00	1.00	1.00
”	Random Error	0.74	0.72	0.71	0.69	0.75	0.72
”	Error‐free	0.73	0.67	0.73	0.64	0.59	0.67
”	Opening Error	0.99	0.98	0.99	1.00	0.99	0.99
”	Closing Error	0.99	0.99	0.98	0.98	0.96	0.98
Sensitivity	Shift Error	1.00	0.98	0.99	0.99	0.97	0.98
”	Random Error	0.92	0.88	0.92	0.88	0.83	0.89
”	Error‐free	0.42	0.42	0.38	0.38	0.51	0.42
”	Opening Error	1.00	1.00	0.99	0.99	0.99	0.99
”	Closing Error	0.94	0.94	0.94	0.93	0.94	0.94
Specificity	Shift Error	1.00	1.00	1.00	1.00	1.00	1.00
”	Random Error	0.91	0.90	0.89	0.89	0.92	0.90
”	Error‐free	0.98	0.97	0.98	0.97	0.96	0.97
”	Opening Error	1.00	0.99	1.00	1.00	1.00	1.00
”	Closing Error	1.00	1.00	0.99	0.99	0.99	0.99
F1‐score	Shift Error	1.00	0.99	0.99	0.99	0.99	0.99
”	Random Error	0.82	0.79	0.80	0.78	0.79	0.80
”	Error‐free	0.54	0.52	0.50	0.48	0.55	0.52
”	Opening Error	1.00	0.99	0.99	1.00	0.99	0.99
”	Closing Error	0.96	0.96	0.96	0.95	0.95	0.96

*Note*: ‘‘Fold 1−5″ refers to the results obtained from each of the five cross‐validation iterations, while “All Folds” indicates the mean value from fold 1 to fold 5.

### 3.2 Classification results between HybridNet‐MC and HybridNet‐SC

**FIGURE 5:**
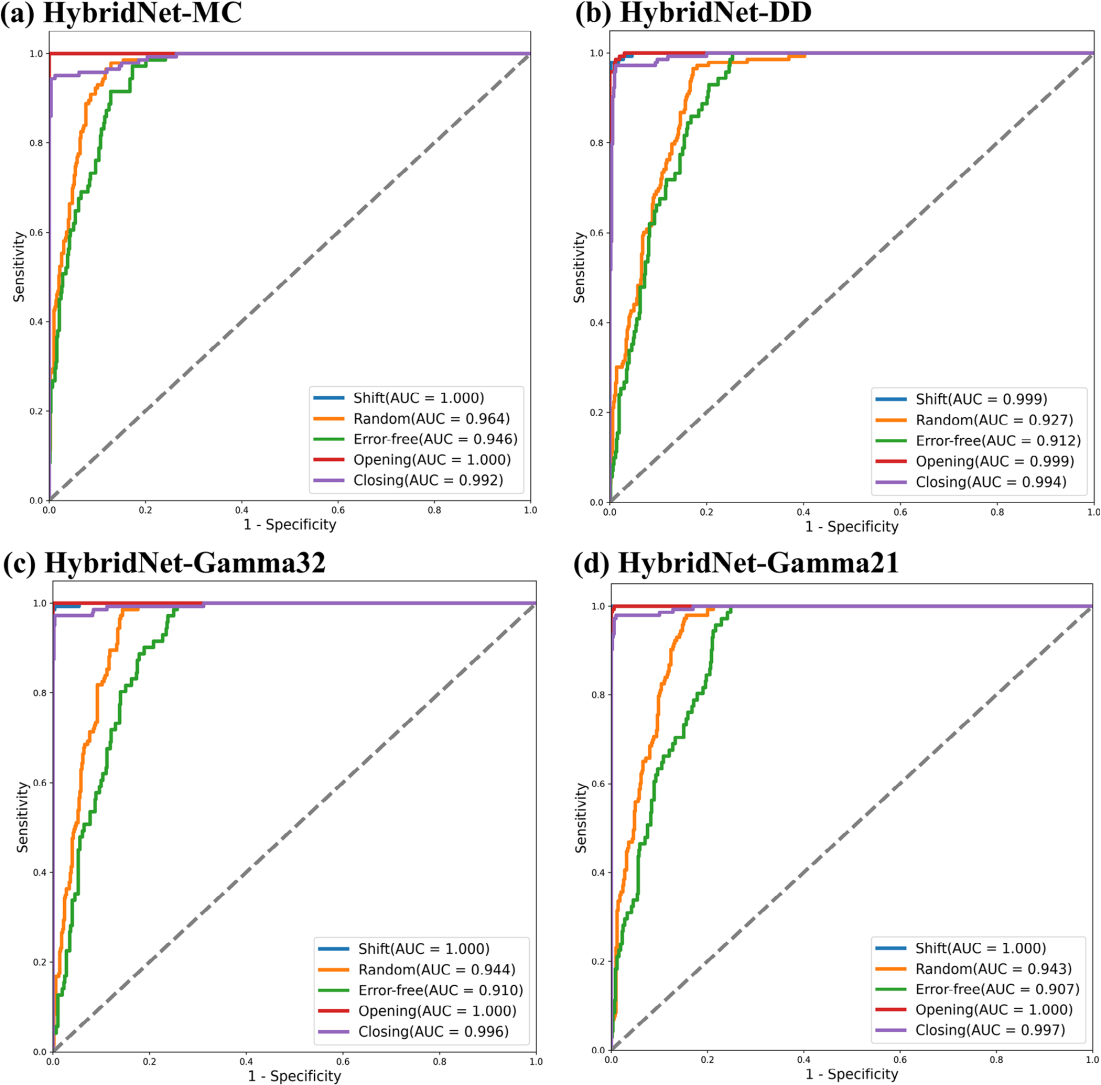
ROC curves of random, shift, error‐free, opening, and closing of four types of networks. (a), (b), (c) and (d) represent HybridNet‐MC, HybridNet‐DD, HybridNet‐Gamma32, and HybridNet‐Gamma21, respectively. ROC, Receiver Operating Characteristic.

**FIGURE 6:**
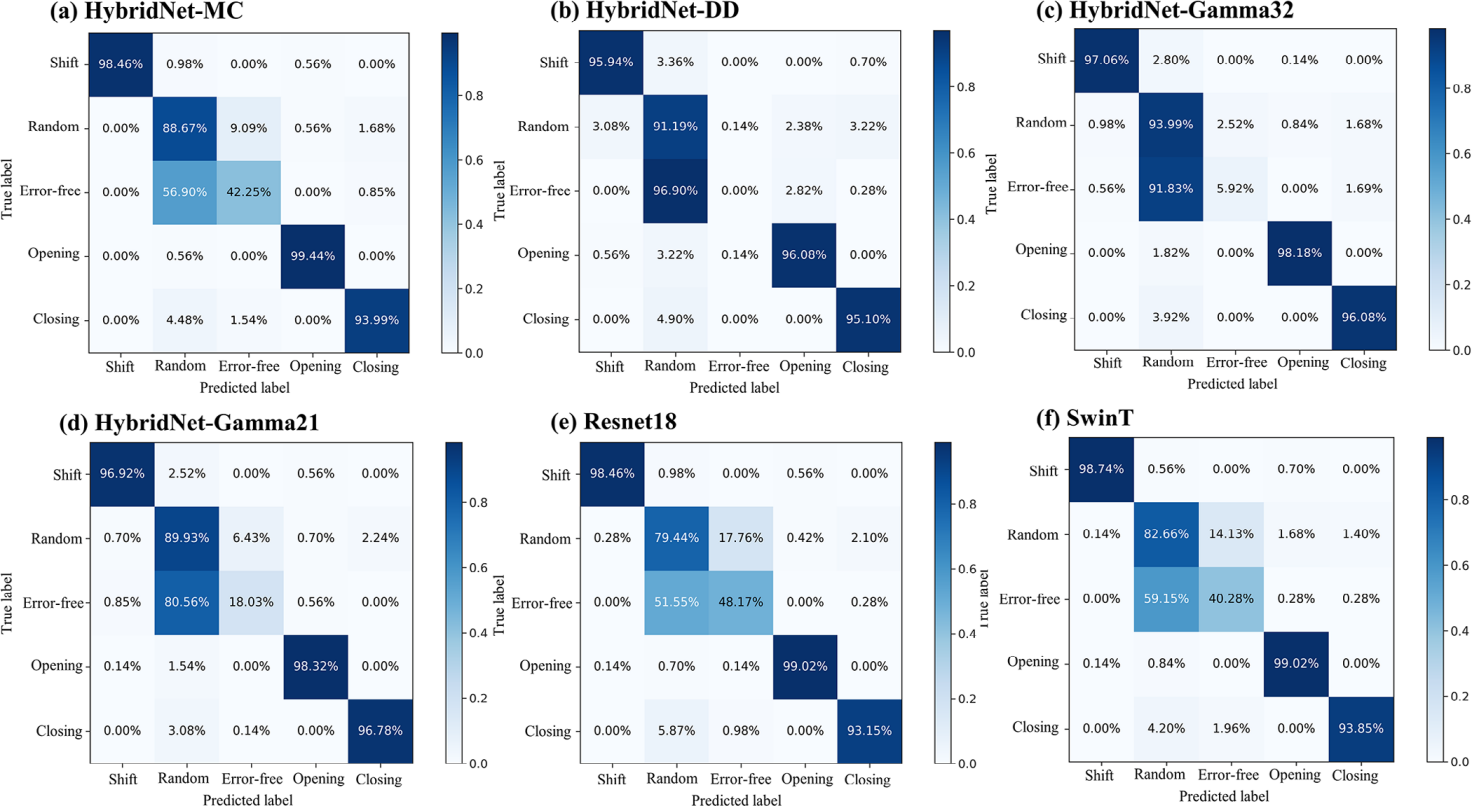
Normalized Confusion Matrices (Averaged over five‐fold Cross‐Validation) for (a) HybridNet‐MC, (b) Hybrid‐DD, (c) HybridNet‐Gamma32, (d) HybridNet‐Gamma21, (e) Resnet‐18 and (f) SwinT Networks. Each matrix represents the relative frequencies of correct classifications and misclassifications among the different classes, providing insights into the performance of the respective networks. The matrices are presented in a normalized format to facilitate comparison and analysis.

A comparative analysis was conducted between the HybridNet‐MC and the HybridNet‐SC trained with DD maps, gamma maps (3%/2 mm), and gamma maps (2%/1 mm), respectively. The results revealed that the HybridNet‐MC performed better in terms of accuracy, precision, sensitivity, specificity, and F1 score than the HybridNet‐SC, as shown in Table 2. Regarding the classification of specific error types, all four networks exhibited similar performance levels for shift errors, opening errors, and closing errors. However, the HybridNet‐MC exhibited enhanced performance compared to the other three single‐channel networks in detecting random errors and error‐free cases, as illustrated in Figures 5 and 6. Specifically, the HybridNet‐MC demonstrated higher AUC values for random errors (0.964) and no errors (0.946) compared to the other three networks.

**TABLE 2:** The precision, accuracy, sensitivity, specificity, and F1‐score of the HybridNet‐MC, Hybrid‐DD, HybridNet‐Gamma32, and HybridNet‐Gamma21 networks were evaluated using five‐fold cross‐validation.

Plan type	Model type	accuracy	precision	sensitivity	specificity	F1‐score
Shift Error	HybridNet‐MC	0.89	1.00	0.98	1.00	0.99
”	Hybridnet‐DD	0.84	0.96	0.96	0.99	0.96
”	HybridNet‐Gamma32	0.87	0.98	0.98	0.99	0.98
”	HybridNet‐Gamma21	0.87	0.99	0.97	1.00	0.98
Random Error	HybridNet‐MC	0.89	0.72	0.89	0.90	0.80
”	Hybridnet‐DD	0.84	0.60	0.91	0.83	0.73
”	HybridNet‐Gamma32	0.87	0.64	0.94	0.85	0.76
”	HybridNet‐Gamma21	0.87	0.66	0.90	0.87	0.76
Error‐free	HybridNet‐MC	0.89	0.67	0.42	0.97	0.52
”	Hybridnet‐DD	0.84	0.00	0.00	1.00	0.00
”	HybridNet‐Gamma32	0.87	0.63	0.08	0.99	0.13
”	HybridNet‐Gamma21	0.87	0.58	0.18	0.98	0.27
Opening Error	HybridNet‐MC	0.89	0.99	0.99	1.00	0.99
”	Hybridnet‐DD	0.84	0.96	0.96	0.99	0.96
”	HybridNet‐Gamma32	0.87	0.99	0.98	1.00	0.98
”	HybridNet‐Gamma21	0.87	0.98	0.98	1.00	0.98
Closing Error	HybridNet‐MC	0.89	0.98	0.94	0.99	0.96
”	Hybridnet‐DD	0.84	0.96	0.95	0.99	0.96
”	HybridNet‐Gamma32	0.87	0.98	0.96	0.99	0.97
”	HybridNet‐Gamma21	0.87	0.98	0.97	0.99	0.97
Average	HybridNet‐MC	**0.89**	**0.87**	**0.85**	**0.97**	**0.85**
”	Hybridnet‐DD	0.84	0.70	0.76	0.96	0.72
”	HybridNet‐Gamma32	0.87	0.84	0.79	0.97	0.77
”	HybridNet‐Gamma21	0.87	0.84	0.80	0.97	0.79

*Note*: Where “Average” signifies the average value obtained across different error types, including shift, random, error‐free, opening, and closing errors.

**FIGURE 7:**
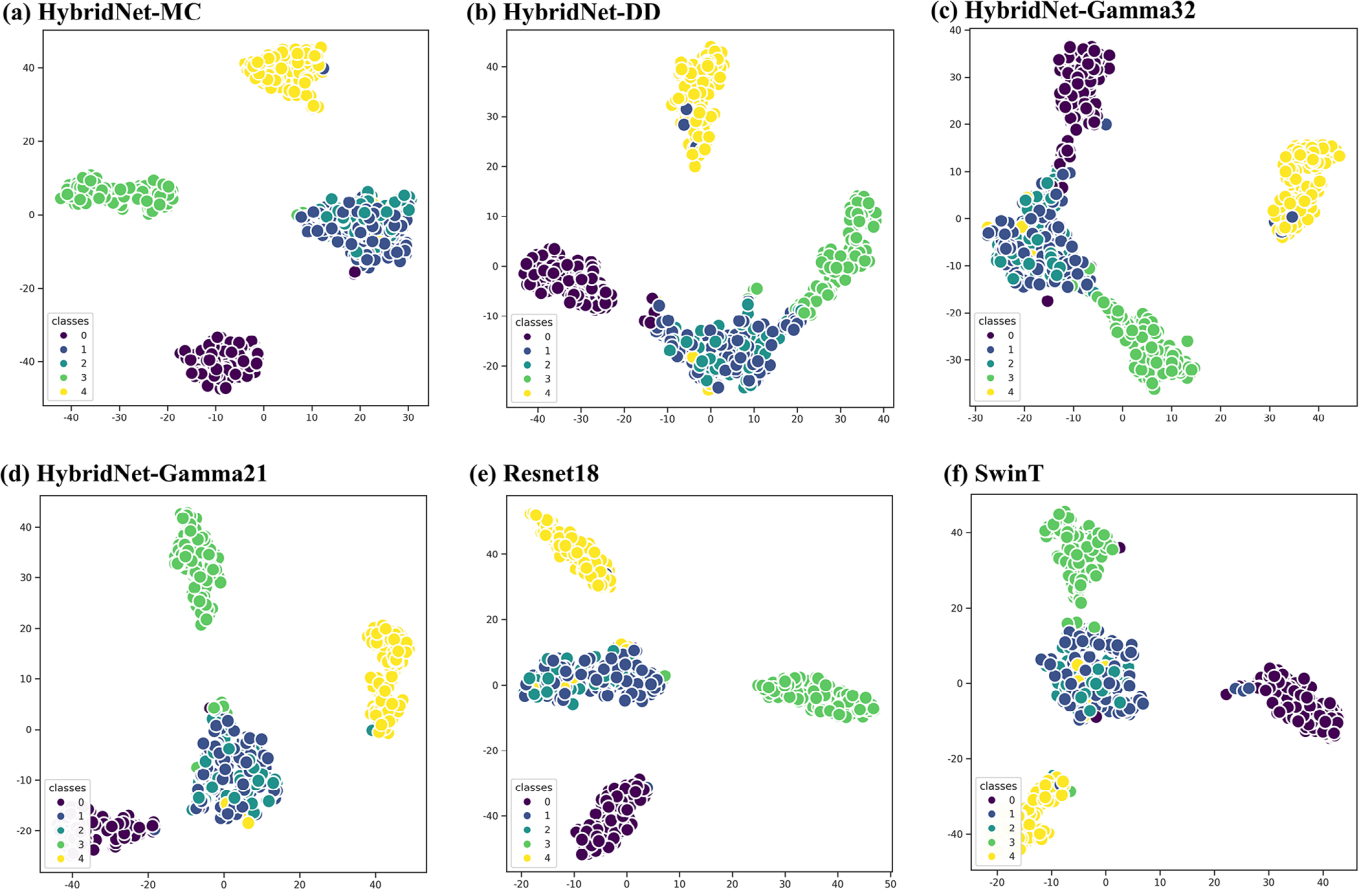
Two‐dimensional scatter plots of input data from four models after dimensionality reduction using t‐SNE (Stochastic Neighbor Embedding), classes 0, 1, 2, 3, and 4 represent shift, random, error‐free, opening, and closing errors, respectively. (a), (b), (c), (d), (e) and (f) represent HybridNet‐MC, HybridNet‐DD, HybridNet‐Gamma32, HybridNet‐Gamma21, Resnet18, and SwinT, respectively.

The high‐dimensional features of Hybrid‐DD, HybridNet‐Gamma32, HybridNet‐Gamma21, and HybridNet‐MC networks were projected onto a two‐dimensional scatter plot using the t‐SNE method, as presented in Figure 7. In comparison to Hybrid‐DD, HybridNet‐Gamma32, and HybridNet‐Gamma21, the HybridNet‐MC network distinctly separated shift errors, opening errors, and closing errors into distinct clusters, with higher separation between clusters and stronger intra‐cluster compactness.

### 3.3 Classification results between HybridNet‐MC and CNN/ transformer networks

The HybridNet‐MC, ResNet‐18, and SwinT were trained using five‐fold cross‐validation on the same training dataset. Compared to the HybridNet‐MC, the average precision of ResNet‐18 and SwinT was 0.86, slightly lower than that of the HybridNet‐MC. When considering the classification of error‐free cases, the precision values for ResNet‐18 and SwinT were 0.56 and 0.57, respectively, demonstrating a significant decrease compared to the HybridNet‐MC's precision value of 0.67. The precision, accuracy, sensitivity, specificity, and F1‐score of the HybridNet‐MC, ResNet‐18, and SwinT are shown in Table 3.

**TABLE 3:** The precision, accuracy, sensitivity, specificity, and F1‐score of the HybridNet‐MC, CNN and transformer networks were evaluated using five‐fold cross‐validation.

Plan type	Model type	accuracy	precision	sensitivity	specificity	F1‐score
Shift Error	HybridNet‐MC	0.89	1.00	0.98	1.00	0.99
”	Resnet18	0.88	1.00	0.98	1.00	0.99
”	SwinT	0.88	1.00	0.99	1.00	0.99
Random Error	HybridNet‐MC	0.89	0.72	0.89	0.90	0.80
”	Resnet18	0.88	0.71	0.79	0.91	0.75
”	SwinT	0.88	0.71	0.83	0.90	0.76
Error‐free	HybridNet‐MC	0.89	0.67	0.42	0.97	0.52
”	Resnet18	0.88	0.56	0.48	0.95	0.52
”	SwinT	0.88	0.57	0.40	0.96	0.45
Opening Error	HybridNet‐MC	0.89	0.99	0.99	1.00	0.99
”	Resnet18	0.88	0.99	0.99	1.00	0.99
”	SwinT	0.88	0.98	0.99	0.99	0.98
Closing Error	HybridNet‐MC	0.89	0.98	0.94	0.99	0.96
”	Resnet18	0.88	0.98	0.93	0.99	0.95
”	SwinT	0.88	0.98	0.94	1.00	0.96
Average	HybridNet‐MC	**0.89**	**0.87**	**0.85**	**0.97**	**0.85**
”	Resnet18	0.88	0.86	0.84	0.97	0.84
”	SwinT	0.89	0.86	0.84	0.97	0.84

*Note*: Where “Average” signifies the average value obtained across different error types, including shift, random, error‐free, opening, and closing errors.

## 4 DISCUSSION

In this paper, we proposed a hybrid network that employed DD maps and gamma maps obtained from a 2D ion chamber array as multi‐channel inputs for detecting MLC errors in patient‐specific IMRT QA. The results demonstrate that the proposed method can effectively identify MLC errors using measured low‐resolution dose maps. Our study built upon previous research by Wootton, Nyflot, and Ma et al., who used EPID to classify MLC error types.^6^, ^7^, ^8^ In our study, we replaced the high‐resolution EPID with a low‐resolution 2D ion chamber array for detecting MLC errors in patient‐specific QA plans. To improve the accuracy of MLC error classification, we utilized multiple types of image inputs and a hybrid network. While our simulation methodology for inducing MLC errors was similar to that of Wootton,^6^ Nyflot,^7^ and Kimura,^9^ the results cannot be directly compared due to the use of different detectors. Nonetheless, our results demonstrated that utilizing multiple types of inputs had higher sensitivity than using a single type of input, and the hybrid network outperformed the CNN network in terms of sensitivity. To the best of our knowledge, this is the first report suggesting that a hybrid network with multiple types of image inputs performs better than a CNN network with a single type of image input for MLC error classification.

Our proposed method achieved the best results on all evaluation metrics when taking merged images as input, as shown in Table 2. The reason behind this is that the merged image has richer semantic information than single‐channel inputs such as DD maps, gamma maps (3%/2 mm), and gamma maps (2%/1 mm). HybridNet can effectively fuse these different types of information and extract high‐quality decision‐making features, resulting in better classification performance. Kimura's research revealed that the precise locations associated with error points can be directly determined using DD maps.^9^ However, our findings indicated that when DD maps were used as stand‐alone input, the classification results became overly sensitive, leading to the potential misclassification of measurement points with significant DDs in error‐free plans as random errors. On the other hand, the use of gamma maps reduced this sensitivity, but the drawback is the lack of direct positional information regarding the error points. For instance, Hybrid‐DD exhibited a sensitivity of 0 for error‐free plans, while HybridNet‐Gamma32 and HybridNet‐Gamma21 achieved a sensitivity of 0.08 and 0.18, respectively. The advantages of a multi‐input network lie in the ability to share information among different types of error images (DD maps, gamma maps) within the feature extraction path, thereby enhancing the performance of the multi‐input network.

The HybridNet exhibited higher discriminability among shift, opening, and closing errors as shown in Figure 7. However, the cluster of random errors overlapped with the cluster of error‐free cases, partially explaining the confusion between random errors and error‐free classes across the four networks. There may be two reasons, the first reason was that the IMRT error plans were simulated in this study by introducing fixed‐value errors to the leaf positions of control points in the MLC. Gaussian errors with standard deviations of 1 and 2 mm were added to the MLC leaf positions to simulate random errors. Due to the small magnitude of MLC leaf position changes and the limited resolution of the detectors, the differences in DD maps or gamma maps between the cluster of random errors and the cluster of error‐free cases were minimal.^33^ The second reason was attributed to the fact that measurement was integrated over time, and errors in different control points may cancel out when summed up. For example, within the same beam, if control point 1 exhibited a positive error at a measurement point and control point 2 showed a corresponding negative error at the same measurement point, the dose error in the beam at that specific measurement point might have diminished or disappeared. These reasons may result in the HybridNet's inability to perfectly differentiate between error‐free and random errors.

As a popular deep learning network, CNN has been widely utilized in various domains such as detection, classification, and segmentation.^34^, ^35^ Transformer was first applied to natural language processing (NLP) as a potential deep learning network and achieved state‐of‐the‐art (SOTA) performance.^36^ The transformer model was recently adopted in computer vision and performed excellently in numerous machine vision tasks.^23^, ^37^ This study employed a hybrid methodology to address the inherent complementarity between local feature extraction and global contextual representation in CNN and ViT. Through a comparative analysis of the HybridNet‐MC network with the conventional CNN and ViT models, the HybridNet‐MC network demonstrated superior network performance, particularly in classification accuracy under random errors and error‐free conditions, as evidenced by the results presented in Table 3. While CNN possess robust capabilities in local feature extraction, it cannot model long‐term dependencies and capture global contextual representations. Conversely, ViT excels at establishing global representations but exhibits limitations in local feature extraction.^38^ Thus, our proposed methodology employed CNN at the network's input stage to extract low‐level features, with a specific emphasis on local feature extraction. Subsequently, ViT was leveraged to model the long‐term dependencies among features, thereby accentuating global contextual relationships. The HybridNet exhibited greater distances between clusters and higher intra‐cluster cohesion, indicating that the hybrid network was more adept at recognizing differences between various MLC error categories and common features among errors within the same category in high‐dimensional feature space, as shown in Figure 7. Thus, the hybrid network architecture allows for the effective integration of the strengths of both CNN and ViT, resulting in enhanced model performance.

In clinical practice, 2D detectors are commonly used in patient‐specific IMRT QA. Due to the lower spatial resolution of 2D detectors and the comparatively insensitivity of gamma analysis, these detectors exhibit insensitivity to detecting MLC leaf position errors. Yan et al. introduced systematic errors ranging from 1 to 2 mm and random errors up to 2 mm in MLC leaf positions during patient‐specific IMRT QA, revealing that only MLC systematic errors of 2 mm or more could be detected.^39^ Shang et al. also found that employing the 2D chamber array could only detect MLC leaf shift errors larger than 2 mm and suggested more sensitive methods to detect subtle MLC errors.^40^ Traditional gamma threshold analysis could not detect these subtle MLC leaf positioning errors, which caused clinically relevant dosimetric changes to planning target volume (PTV) and organs at risk (OAR) of treatment plans. Bai et al. recommended controlling MLC leaf random errors to below 2 mm and systematic errors within 0.5 mm to minimize dose changes in the clinical relevance of the PTV and OAR for nasopharyngeal carcinoma.^41^ In previous studies,^6^, ^7^, ^8^, ^9^ deep learning methods enhanced sensitivity in detecting MLC positioning errors compared to traditional gamma analysis. These studies focused on EPID or 3D detectors, lacking application to 2D low‐resolution detectors. To improve the detection sensitivity and classification accuracy of MLC positioning errors with 2D low‐resolution detectors, we employed a hybrid deep network architecture combining CNN and Vit, along with a multi‐channel input using DD and Gamma maps. The results demonstrated classification accuracy exceeding 0.8 for systematic and random errors ranging from 1 to 2 mm. Compared to the studies by Yan^39^ and Shang,^40^ our proposed method effectively enhanced the sensitivity and classification accuracy of MLC positioning error detection. This work advanced the sensitivity of low‐resolution detectors in detecting MLC positioning errors and enabled the classification of MLC positioning error types, thereby ensuring delivery accuracy.

Our study was subject to several limitations that should be noted. First, our investigation focused on classifying MLC leaf position errors using a low‐resolution 2D ion chamber detector in patient‐specific QA. Although MLC leaf position errors represented a typical and well‐recognized type of error, it was important to acknowledge the existence of other error sources within patient‐specific QA, such as MU, collimator rotation, gantry rotation, transmission factor, etc.^42^ Our study aimed to detect and classify MLC positioning errors. The detection and classification of other MLC errors, such as leaf transmission, jaw tracking positions, and multi‐MLC errors combined, will be subjects in our future research. Another limitation pertained to the narrow focus of our method, which solely encompassed the classification of MLC errors without considering their impact on clinically relevant dose distributions within the PTV or OAR regions. Specifically, our method did not assess whether MLC errors introduced notable deviations in dose distribution within these critical anatomical structures. If the errors resulted in dose discrepancies in clinically insignificant regions, they might be deemed clinically acceptable. To address this limitation in future research, we propose to incorporate the delineation of PTV and OAR contours as additional input variables. By augmenting the existing classification framework, we aim to simultaneously assess their adherence to the requirements of clinically relevant dose distributions.

## 5 CONCLUSION

In this study, we have developed a novel multi‐channel hybrid network that combines CNN with Vit to address the detection of errors in the MLC for patient‐specific IMRT QA. Our approach involves a comprehensive analysis of multi‐channel DD maps and gamma maps generated from low‐resolution two‐dimensional dose matrices. Notably, the hybrid model trained on these maps exhibits a high level of accuracy in identifying MLC errors, surpassing models trained solely on single channel DD maps or gamma maps. Furthermore, when compared to conventional CNN and transformer models, our hybrid model demonstrates superior precision in detecting MLC errors, highlighting its unique advantages in IMRT QA. By harnessing the combined capabilities of multiple‐channel DD maps and gamma maps, our approach offers an effective and reliable solution for detecting MLC errors in IMRT QA processes, especially when employing low‐resolution 2D ion chamber array. Future research can further explore the potential of our hybrid model in clinical applications, ultimately enhancing the overall quality and safety of IMRT treatments.

## AUTHOR CONTRIBUTIONS

Bing Yan conceived the experiments. Bing Yan and Jun Shi acquired and analyzed the data for the work. Bing Yan, Jun Shi, Hu Peng, Aidong Wu and Xiao Wang designed the study and analyzed the result. Bing Yan, Xudong Xue, Hu Peng, Jun Shi and Chi Ma participated in writing manuscript. The final version of the manuscript has been reviewed and approved for publication by all authors.

## CONFLICT OF INTEREST STATEMENT

The authors have no conflicts to disclose.

## References

[1] Group IMRTCW . Intensity‐modulated radiotherapy: current status and issues of interest. Int J Radiat Oncol Biol Phys. 2001;51(4):880‐914. doi:10.1016/s0360-3016(01)01749-7 11704310

[2] James HV , Scrase CD , Poynter AJ . Practical experience with intensity‐modulated radiotherapy. Br J Radiol. 2004;77(913):3‐14. doi:10.1259/bjr/14996943 14988132

[3] Palta JR , Liu C , Li JG . Quality assurance of intensity‐modulated radiation therapy. Int J Radiat Oncol Biol Phys. 2008;71(1):S108‐S112. doi:10.1016/j.ijrobp.2007.05.092 Suppl 18406906

[4] Miften M , Olch A , Mihailidis D , et al. Tolerance limits and methodologies for IMRT measurement‐based verification QA: recommendations of AAPM Task Group No. 218. Med phys. 2018;45(4):e53‐e83. doi:10.1002/mp.12810 29443390

[5] Nelms BE , Chan MF , Jarry G , et al. Evaluating IMRT and VMAT dose accuracy: practical examples of failure to detect systematic errors when applying a commonly used metric and action levels. Med phys. 2013;40(11):111722. doi:10.1118/1.4826166 24320430 PMC8353583

[6] Wootton LS , Nyflot MJ , Chaovalitwongse WA , Ford E . Error detection in intensity‐modulated radiation therapy quality assurance using radiomic analysis of gamma distributions. Int J Radiat Oncol Biol Phys. 2018;102(1):219‐228. doi:10.1016/j.ijrobp.2018.05.033 30102197

[7] Nyflot MJ , Thammasorn P , Wootton LS , Ford EC , Chaovalitwongse WA . Deep learning for patient‐specific quality assurance: identifying errors in radiotherapy delivery by radiomic analysis of gamma images with convolutional neural networks. Med Phys. 2019;46(2):456‐464. doi:10.1002/mp.13338 30548601

[8] Ma C , Wang R , Zhou S , et al. The structural similarity index for IMRT quality assurance: radiomics‐based error classification. Med Phys. 2021;48(1):80‐93. doi:10.1002/mp.14559 33128263

[9] Kimura Y , Kadoya N , Tomori S , Oku Y , Jingu K . Error detection using a convolutional neural network with dose difference maps in patient‐specific quality assurance for volumetric modulated arc therapy. Phys Med. 2020;73:57‐64. doi:10.1016/j.ejmp.2020.03.022 32330812

[10] Pan Y , Yang R , Zhang S , et al. National survey of patient specific IMRT quality assurance in China. Radiat Oncol. 2019;14(1):69. doi:10.1186/s13014-019-1273-5 31023348 PMC6482589

[11] Lam D , Zhang X , Li H , et al. Predicting gamma passing rates for portal dosimetry‐based IMRT QA using machine learning. Med Phys. 2019;46(10):4666‐4675. doi:10.1002/mp.13752 31386761

[12] Liang B , Liu B , Zhou F , Yin FF , Wu Q . Comparisons of volumetric modulated arc therapy (VMAT) quality assurance (QA) systems: sensitivity analysis to machine errors. Radiat Oncol. 2016;11(1):146. doi:10.1186/s13014-016-0725-4 27821135 PMC5100111

[13] Wolfs CJA , Verhaegen F . What is the optimal input information for deep learning‐based pre‐treatment error identification in radiotherapy? Phys Imaging Radiat Oncol. 2022;24:14‐20. doi:10.1016/j.phro.2022.08.007 36106060 PMC9465434

[14] Byrnes K , Ford A , Bennie N . Verification of the Elekta Monaco TPS Monte Carlo in modelling radiation transmission through metals in a water equivalent phantom. Australas Phys Eng Sci Med. 2019;42(2):639‐645. doi:10.1007/s13246-019-00749-2 30863988

[15] Kimura Y , Kadoya N , Oku Y , Kajikawa T , Tomori S , Jingu K . Error detection model developed using a multi‐task convolutional neural network in patient‐specific quality assurance for volumetric‐modulated arc therapy. Med Phys. 2021;48(9):4769‐4783. doi:10.1002/mp.15031 34101848

[16] Mohammadi M , Rostampour N , Rutten TP . Modification of the gamma function for the recognition of over‐ and under‐dose regions in three dimensions. J Med Phys. 2012;37(4):200‐206. doi:10.4103/0971-6203.103605 23293451 PMC3532748

[17] Liu Z , Lin Y , Cao Y , et al. Swin transformer: Hierarchical vision transformer using shifted windows. arXiv preprint arXiv: 2103.14030. 2021. doi:10.48550/arXiv.2103.14030

[18] He K , Zhang X , Ren S , Sun J . Deep residual learning for image recognition. In: 2016 IEEE Conference on Computer Vision and Pattern Recognition (CVPR). IEEE; 2016:770‐778. doi:10.1109/CVPR.2016.90

[19] Hu J , Shen L , Albanie S , Sun G , Wu E . Squeeze‐and‐excitation networks. arXiv preprint arXiv: 1709.01507. 2017. doi:10.48550/arXiv.1709.01507 31034408

[20] Huang G , Liu Z , Maaten L , Weinberger KQ . Densely connected convolutional networks. arXiv preprint arXiv: 1608.06993. 2016. doi:10.48550/arXiv.1608.06993

[21] Tan M , Le Q . Efficientnet: Rethinking model scaling for convolutional neural networks. arXiv preprint arXiv: 1905.11946. 2019. doi:10.48550/arXiv.1905.11946

[22] Radosavovic I , Kosaraju RP , Girshick R , He K , Dollar P . Designing network design spaces. arXiv preprint arXiv: 2003.13678. 2020. doi:10.48550/arXiv.2003.13678

[23] Vaswani A , Shazeer N , Parmar N , et al. Attention is all you need. Adv Neural Inf Process Syst. 2017;30:6000–6010. doi:10.5555/3295222.3295349

[24] Dosovitskiy A , Beyer L , Kolesnikov A , et al. An Image is Worth 16×16 Words: Transformers for Image Recognition at Scale. arXiv preprint arXiv:2010.11929. 2020.

[25] Loshchilov I , Hutter F . Decoupled Weight Decay Regularization. arXiv preprint arXiv:1711.05101. 2017. doi:10.48550/arXiv.1711.05101

[26] Loshchilov I , Hutter F . Sgdr: Stochastic gradient descent with warm restarts. arXiv preprint arXiv:1608.03983. 2016. doi:10.48550/arXiv.1608.03983

[27] Zhong Z , Zheng L , Kang G , et al. Random erasing data augmentation. arXiv preprint arXiv:1708.04896. doi:10.48550/arXiv.1708.04896

[28] Nakamura S , Sakai M , Ishizaka N , et al. Deep learning‐based detection and classification of multi‐leaf collimator modeling errors in volumetric modulated radiation therapy. J Appl Clin Med Phys. 2023;24(12):e14136. doi:10.1002/acm2.14136 37633834 PMC10691639

[29] Alomar K , Aysel HI , Cai X . Data augmentation in classification and segmentation: a survey and new strategies. J Imaging. 2023;9(2):46. doi:10.3390/jimaging9020046 36826965 PMC9966095

[30] Feng B , Zhou W , Yang X , et al. Pseudo‐siamese network combined with dosimetric and clinical factors, radiomics features, CT images and 3D dose distribution for the prediction of radiation pneumonitis: a feasibility study. Clin Transl Radiat Oncol. 2022;38:188‐194. doi:10.1016/j.ctro.2022.11.011. Published 2022 Nov 22. 36479235 PMC9720487

[31] Fawcett T . An introduction to ROC analysis. Pattern Recognit Lett. 2006;27(8):861‐874. doi:10.1016/j.patrec.2005.10.010

[32] Van der Maaten L , Hinton G . Visualizing data using t‐SNE. J Mach Learn Res. 2008;9(11):2579‐2605. https://www.jmlr.org/papers/v9/vandermaaten08a.html

[33] Hussein M , Adams EJ , Jordan TJ , Clark CH , Nisbet A . A critical evaluation of the PTW 2D‐ARRAY seven29 and OCTAVIUS II phantom for IMRT and VMAT verification. J Appl Clin Med Phys. 2013;14(6):274‐292. doi:10.1120/jacmp.v14i6.4460 PMC5714639 24257288

[34] Minaee S , Luo P , Lin Z , Bowyer K . Going deeper into face detection: A survey. arXiv preprint arXiv:2103.14983. 2021. doi:10.48550/arXiv.2103.14983

[35] Minnema J , Ernst A , van Eijnatten M , et al. A review on the application of deep learning for CT reconstruction, bone segmentation and surgical planning in oral and maxillofacial surgery. Dentomaxillofac Radiol. 2022;51(7):20210437. doi:10.1259/dmfr.20210437 35532946 PMC9522976

[36] Gillioz A , Casas J , Mugellini E , Khaled OA . Overview of the Transformer‐based Models for NLP Tasks. In: 15th Conference on Computer Science and Information Systems (FedCSIS). IEEE; 2020:179‐183. doi:10.15439/2020F20

[37] Devlin J , Chang M‐W , Lee K , Toutanova K , Bert: Pre‐training of deep bidirectional transformers for language understanding. arXiv preprint arXiv:1810.04805. 2018. doi:10.48550/arXiv.1810.04805

[38] Liu W , Li C , Xu N , et al. CVM‐Cervix: a hybrid cervical Pap‐smear image classification framework using CNN, visual transformer and multilayer perceptron. Pattern Recognit. 2022;130:108829. doi:10.1016/j.patcog.2022.108829

[39] Yan G , Liu C , Simon TA , Peng LC , Fox C , Li JG . On the sensitivity of patient‐specific IMRT QA to MLC positioning errors. J Appl Clin Med Phys. 2009;10(1):120‐128. doi:10.1120/jacmp.v10i1.2915 PMC5720508 19223841

[40] Shang Q , Godley A , Huang L , Qi P , Xia P . Sensitivity of array detector measurements in determining shifts of MLC leaf positions. J Appl Clin Med Phys. 2017;18(5):80‐88. doi:10.1002/acm2.12148 28799273 PMC5874934

[41] Bai S , Li G , Wang M , Jiang Q , Zhang Y , Wei Y . Effect of MLC leaf position, collimator rotation angle, and gantry rotation angle errors on intensity‐modulated radiotherapy plans for nasopharyngeal carcinoma. Med Dosim. 2013;38(2):143‐147. doi:10.1016/j.meddos.2012.10.002 23402928

[42] Klein EE , Hanley J , Bayouth J , et al. Task Group 142 report: quality assurance of medical accelerators. Med phys. 2009;36(9Part1):4197‐4212. doi:10.1118/1.3190392 19810494

